# Evaluation of Chest CT Scan Findings in Pediatric Patients With COVID‐19: A Retrospective Descriptive Study

**DOI:** 10.1002/hsr2.71532

**Published:** 2025-11-20

**Authors:** Mohammad Hadi Gharib, Dayan Amanian, Reza Zahedpasha

**Affiliations:** ^1^ School of Medicine, 5th Azar Hospital Golestan University of Medical Sciences Gorgan Iran; ^2^ Department of Radiology McMaster University Hamilton Canada; ^3^ Department of Radiology, School of Medicine, 5th Azar Hospital Golestan University of Medical Sciences Gorgan Iran; ^4^ Tu Lab for Diagnostic Research Yale School of Medicine New Haven Connecticut USA

**Keywords:** COVID‐19, diagnosis, pediatrics, tomography, X‐ray computed

## Abstract

**Background and Aims:**

COVID‐19 in children presents with varying severity. Identifying characteristic chest CT features is essential for accurate diagnosis and screening. This study aimed to evaluate CT patterns in pediatric COVID‐19 cases to enhance diagnostic accuracy.

**Methods:**

This retrospective cross‐sectional study analyzed chest CT scans of 42 children with confirmed COVID‐19 at Children's Hospital, Gorgan, Iran. A 14‐item checklist assessed demographics, lung involvement, and radiological features. Descriptive statistics and Fisher's exact test were used for analysis.

**Results:**

Ground‐glass opacities (GGO) were the most common finding (92.9%), followed by consolidation (54.8%). Both were significantly associated with peripheral distribution (*p* < 0.001) and lower zone involvement (*p* < 0.001 for GGO, *p* ≈ 0.002 for consolidation). Lesions affected peripheral lung zones (45.24%) or both central and peripheral zones (40.48%), with consolidation predominantly in the latter (*p* < 0.001). Notably, 7.1% of children had no visible lung lesions. Cases with < 25% lung involvement showed significant correlation with GGO (*p* < 0.001). Pleural effusion was observed in 4.8%, while pericardial effusion and mediastinal lymphadenopathy were absent.

**Conclusion:**

Pediatric COVID‐19 commonly presents with GGO, consolidation, and peripheral lung lesions, with rare occurrence of features such as pleural effusion compared to adults. These differences may refine diagnostic strategies for pediatric populations.

## 1 Introduction

The novel coronavirus (2019‐nCoV), now known as COVID‐19, was first identified in Wuhan, China, in December 2019 and rapidly spread to other regions. Coronaviruses are known to cause a range of illnesses from the common cold to severe respiratory conditions such as SARS and MERS [1, 2]. SARS‐CoV‐2 enters host cells via ACE2 receptors on respiratory epithelial cells, with an average incubation period of 5−6 days. In children, this incubation phase tends to be shorter than in adults, averaging around 2 days [3]. Approximately 2.5%−18% of individuals may remain asymptomatic and can transmit the virus even before symptoms develop [4, 5].

Initially, it was believed that children were less affected by COVID‐19, displaying either mild symptoms or remaining asymptomatic. However, as the pandemic progressed, severe cases in children were also reported [6, 7]. Clinical manifestations of COVID‐19 in children tend to be milder compared to adults, ranging from asymptomatic infections to severe respiratory involvement [8]. While children commonly present with mild symptoms such as rhinorrhea, sneezing, and coughing, some may progress to severe respiratory distress or failure [9]. This may be due to lower expression of ACE2 receptors in children [10, 11].

Accurate diagnosis of COVID‐19 in children is crucial. Standard diagnostic methods include reverse transcription‐polymerase chain reaction (RT‐PCR) and computed tomography (CT) imaging. CT scans are frequently used to diagnose various forms of pneumonia, including COVID‐19. Chest CT imaging provides valuable information through radiographic features such as patchy consolidation and multifocal ground‐glass opacities (GGO) or interstitial changes with a peripheral distribution. These findings are instrumental in assessing lung inflammation and predicting the prognosis of COVID‐19 [12, 13, 14, 15]. Atypical CT findings, though rare, may suggest concurrent bacterial pneumonia or alternative diagnoses. These include mediastinal lymphadenopathy, pleural effusions, multiple pulmonary nodules, the “tree‐in‐bud” sign, pneumothorax, cavitation, the “atoll sign,” and pneumomediastinum [16, 17, 18]. Identifying these uncommon features should prompt evaluation for co‐infections or alternative conditions beyond the typical COVID‐19 presentation.

The chest CT images demonstrate a broad spectrum of pulmonary involvement in pediatric COVID‐19 patients. These images illustrate the progression of radiological findings from early to advanced stages of the disease. Initially, air trapping is observed, characteristic of viral bronchiolitis. As the disease progresses, GGO appear, indicating the spread of inflammation to the alveoli. In more advanced stages, consolidation ranging from mild to severe is seen, reflecting more extensive lung tissue involvement. This progressive pattern shows that pulmonary involvement extends from the bronchiolar walls into the lung tissue, with increasing density of involvement.

This study aims to delineate a specific pattern of chest CT imaging findings in pediatric COVID‐19 patients admitted to the Children's Hospital in Gorgan during 2022. The findings will contribute to the development of more effective strategies for managing and controlling COVID‐19 in children.

## 2 Materials and Methods

This cross‐sectional, descriptive‐analytical, and retrospective study was conducted to analyze chest CT scan findings in pediatric COVID‐19 patients. The study adhered to ethical guidelines, and all necessary permissions were obtained before its commencement. This study was approved by the Ethics Committee of Golestan University of Medical Sciences (Ethics Code: IR.GOUMS.REC.1403.047). Given the retrospective nature of the study, the requirement for informed consent was waived by the Ethics Committee. The study population comprised all patients hospitalized with COVID‐19 symptoms and positive RT‐PCR tests at the Children's Hospital during 2021 and 2022. A census sampling method was used, resulting in the inclusion of 42 patients.

The inclusion criteria for the study were hospitalization with COVID‐19 symptoms, a positive RT‐PCR test, and the performance of a chest CT scan at the Children's Hospital during the specified period. Since this was a retrospective study with a census approach, no specific exclusion criteria were applied.

Data were collected from the medical records of the relevant patients. All chest CT scans were reviewed by a radiologist with over 10 years of experience, holding an MD degree and a Pediatric Radiology Fellowship, using a predefined checklist. The data collected included patient demographics, lung zone involvement (upper, middle, lower), presence of GGO, consolidation, the number and type of lung lobes affected, and the presence of pleural or pericardial effusion, lymphadenopathy, and other abnormalities such as cavitation, interlobular septal thickening, and lung involvement volume. CT scans were performed in the supine position without contrast using a PHILIPS (MX 16‐slice) scanner with specific imaging parameters. All chest CT scans were performed using a pediatric protocol to minimize radiation. Tube voltage was set at 80–100 kVp with automatic exposure control. Slice thickness was 1–1.5 mm. Scans covered the entire lungs from apex to diaphragm. The breath‐hold technique was used when possible; otherwise, free breathing was allowed. No routine intravenous contrast was given.

Visual semi‐quantitative assessment of lung involvement was performed based on estimated volumetric extent, following the categorization used by Alireza Aziz‐Ahari et al. [19], and classified into four groups: < 25%, 25%–49%, 50%–74%, and > 75% involvement [20, 21].

For data analysis, the collected data were entered into IBM SPSS Statistics, version 23. Descriptive statistics, including mean and standard deviation, were used for quantitative variables, while qualitative variables were analyzed using frequency distribution tables. Fisher's exact test was employed for statistical analysis, and a *p* < 0.05 was considered statistically significant. The normality of the data was assessed using the Shapiro−Wilk test, and parametric tests were applied accordingly.

## 3 Result

In this study, the chest CT scan findings of 42 pediatric patients hospitalized at the Children's Hospital in Gorgan were analyzed. All patients had positive RT‐PCR tests for COVID‐19, and their CT scans were performed between 0 and 7 days after the onset of symptoms. All children were hospitalized in general wards, with none requiring admission to the ICU. Furthermore, none of the patients had any underlying or congenital conditions. Among these patients, 22 (52.38%) were male and 20 were female. The average age of the patients was 8.26 years with a standard deviation of 3.12 years. The ages ranged from a minimum of 3 years to a maximum of 15 years.

### 3.1 Chest CT Evaluation

**Figure 1:**
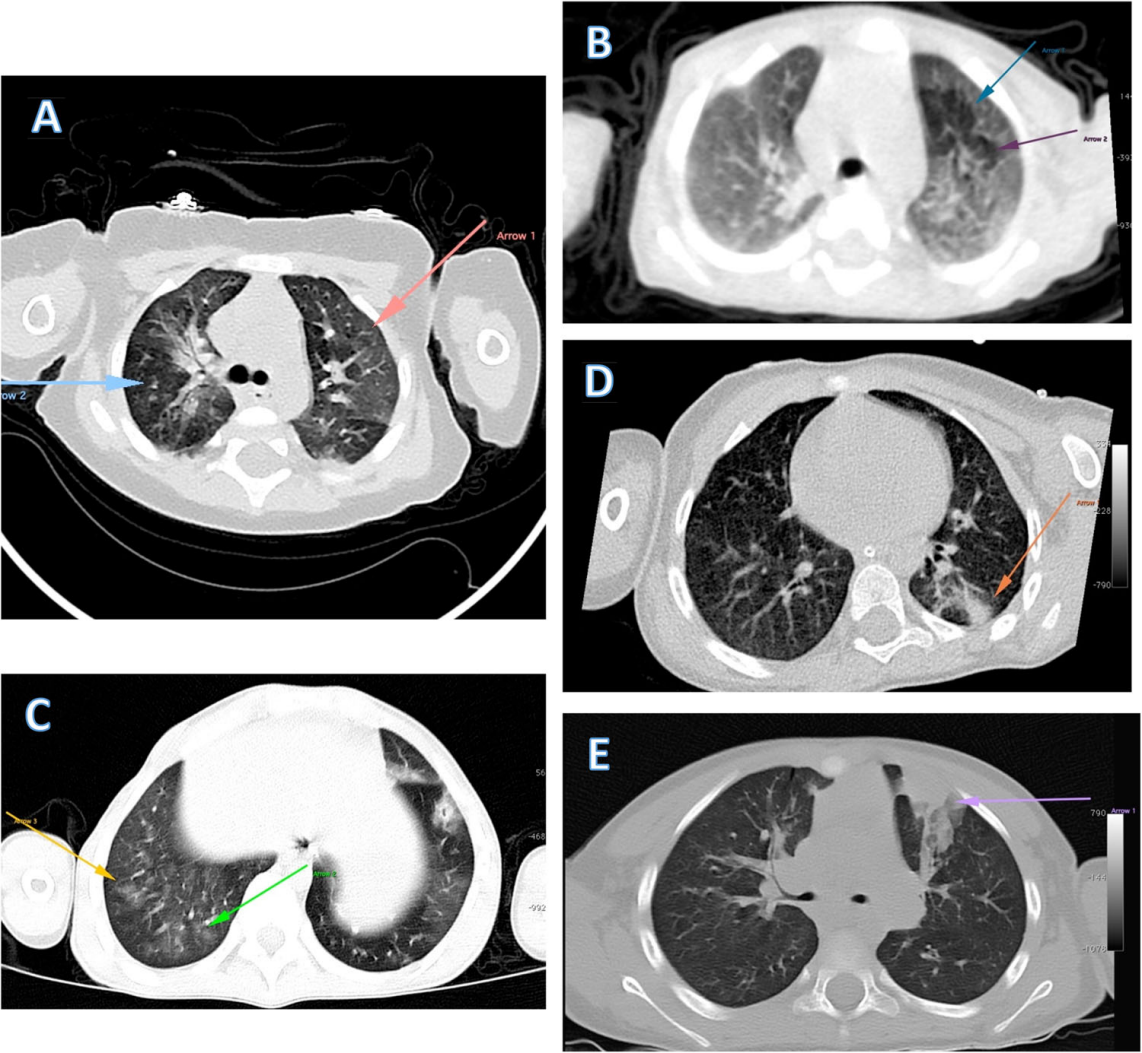
Key findings from different pediatric COVID‐19 patients with lung CT findings. In the first row, images (A) and (B) display prominent air trapping, particularly in image (B), suggesting severe underlying bronchiolitis. In image (C), we observe early stages of ground‐glass opacity in the right lower lobe of the right lung. Images (D) and (E) respectively depict early and significant consolidation in two different patients with COVID‐19 infection.

The chest CT scan findings revealed several key patterns in the pediatric patients with COVID‐19. GGO and consolidation were observed in the majority of cases. Notably, 7.1% of the children (three patients) had no detectable lung lesions. Among those with lung involvement, peripheral lung lesions were the most common, observed in 43.9% of the cases, followed by a combination of central and peripheral lesions in 41.5% of the patients. The most frequently affected lobes were the lower lobes (Figure 1). Pleural effusion was not commonly observed in these patients. The lower lung zones were the most frequently affected, with 69% of cases showing involvement in this area. Approximately one‐third of the children had involvement of three lung lobes.

To facilitate a clearer understanding of these findings, a summary of the results is provided in Tables 1, 2, 3.

**Table 1:** Comparison of radiological findings in pediatric COVID‐19: Data from our study and other published studies.

Our study		Other studies							
Feature	Number of patients (%)	Najafinejad et al. [19]	Ghodsi et al. [20]	Ma et al.[21]	Shelmerdine et al.[22]	Bayramoglu et al. [23]	Palabiyik et al.[24]	Kumar et al.[25]	Yang et al.[26]
Ground‐glass opacities									
Yes	39 (92.9%)	100%	71.4%	67%	62.4%	46%	41%	40%	12.1%
Consolidation									
Yes	23 (54.8%)	64%	28.57%	37%	—	16.2%	36%	67%	8.2%
Lung zone involvement									
No involvement	3 (7.1%)	0%		14%	34%	—	—	33%	11.4%
Upper zone	1 (2.4%)	—	—	—	17.2%	—	—	—	—
Lower zone	29 (69.0%)	—	—	65%	44.4%	—	69%	—	—
Entire lung	9 (21.4%)	—	—	—	4.4%	—	—	—	—
Lesion type									
Central	3 (7.14%)	—	—	—	—	—	—	—	—
Peripheral	19 (45.24%)	—	—	95%	—	More peripheral	—	—	More peripheral
Both (central and peripheral)	17 (40.48%)	—	—	—	—	—	55%	—	—
No lesion	3 (7.14%)	—	—	—	—	—	—	—	—

**Table 2:** Distribution of lung involvement among patients, including the most commonly affected lobes, number of affected lobes per patient, and extent of lung involvement volume.

Most common affected lobes	Number of patients (%)
Left lower lobe (LL)	15 (38.1%)
Left superior lobe (LS)	4 (9.5%)
Right lower lobe (RL)	11 (26.2%)
Right middle lobe (RM)	5 (11.9%)
Right superior lobe (RS)	4 (9.5%)
No involvement	3 (4.8%)
Number of affected lobes	
Zero	3 (7.1%)
1	4 (9.5%)
2	11 (26.2%)
3	12 (28.6%)
4	7 (16.7%)
5	5 (11.9%)
Lung involvement volume	
Zero	3 (7.1%)
Less than 25%	20 (47.6%)
25%−50%	15 (35.7%)
50%−75%	3 (7.1%)
More than 75%	1 (2.4%)

**Table 3:** Rare clinical findings observed in the study population, including pleural effusion, pericardial effusion, mediastinal lymphadenopathy, liver lesions, and bone lesions, with their respective frequencies.

Finding	Number of patients (%)
Pleural effusion	2 (4.8%)
Pericardial effusion	0 (0%)
Mediastinal lymphadenopathy	0 (0%)
Liver lesions	0 (0%)
Bone lesions	0 (0%)

Statistical analysis revealed significant associations between radiological findings and disease patterns, as shown in Table 4. Lower zone involvement demonstrated strong statistical significance for both ground glass opacities (*p* < 0.001) and consolidation (*p* ≈ 0.002). Peripheral lesion distribution showed highly significant associations for both ground glass opacities and consolidation (*p* < 0.001 for both). Notably, lung involvement volume less than 25% was significantly associated with ground glass opacities (*p* < 0.001), while entire lung involvement showed a significant correlation with consolidation (*p* < 0.001). Both central and peripheral lesion patterns (“Both” category) demonstrated significant association with consolidation (*p* < 0.001).

**Table 4:** Association between radiological patterns, zone involvement, lesion types, and lung involvement volumes.

Variable	*p* value (ground glass)	*p* value (consolidation)
Zone involvement (no involvement)	N/A	N/A
Zone involvement (upper zone)	*p* > 0.99	*p* > 0.99
Zone involvement (lower zone)	*p* = 0.008	*p* = 0.008
Zone involvement (entire lung)	*p* = 0.05	*p* = 0.008
Lesion type (doesn′t have)	N/A	N/A
Lesion type (central)	*p* = 0.054	*p* = 0.106
Lesion type (peripheral)	*p* < 0.001	*p* < 0.001
Lesion type (both)	*p* = 0.054	*p* < 0.001
Lung involvement volume (no involvement)	N/A	N/A
Lung involvement volume (< 25%)	*p* < 0.001	*p* = 0.134
Lung involvement volume (25%−50%)	*p* = 0.054	*p* > 0.99
Lung involvement volume (50%−75%)	*p* = 0.054	*p* > 0.99
Lung involvement volume (> 75%)	*p* > 0.99	*p* > 0.99

*Note: p* values were calculated to assess the significance of ground glass opacities and consolidation patterns in chest CT scans.

Abbreviation: N/A, not applicable.

## 4 Discussion

In this retrospective study, which evaluated 42 pediatric patients, the sample size was notably larger compared to previous original studies. The analysis focused on patients with chest CT scans. The findings revealed that GGO and consolidations were among the most common lesions observed. Additionally, involvement was predominantly in the lower lung zones. This larger cohort and detailed imaging analysis provide a more comprehensive understanding of the radiological manifestations of pediatric COVID‐19, highlighting both the prevalence of specific types of pulmonary lesions and the predominant patterns of lung involvement.

GGO was the predominant finding, present in 92.9% of cases. Statistical analysis revealed significant associations between GGO and lower zone involvement (*p* < 0.001) as well as peripheral distribution (*p* < 0.001). This high prevalence is consistent with some previous studies, such as Najafinejad et al. [21]'s work at the same hospital, which reported GGO 100% in all CT‐scanned children, including those in the ICU. However, other studies have reported lower rates, ranging from 40% to 71.43% [22, 23, 24, 25, 26, 27]. The variability in GGO prevalence across studies may be attributed to differences in patient populations, disease severity, and timing of CT scans relative to symptom onset. Notably, Yang et al. [28] reported a much lower incidence of GGO at around 12.1%, likely due to their inclusion of patients who underwent CT scans following a positive RT‐PCR result, regardless of symptom severity. Additionally, in adult populations, the prevalence of GGO has been reported to range between 50% and 80% [29, 30, 31].

Lung consolidation was observed in 54.8% of patients, showing significant correlation with entire lung involvement (*p* < 0.001) and peripheral distribution (*p* < 0.001). This figure falls within the range reported by other researchers [21, 22, 23, 25, 26, 27]. In adult populations, consolidation has been reported in approximately 24%−44% of cases, and consolidation alone without GGO has been reported in about 32% of cases [29, 32]. The wide variation in consolidation rates across studies underscores the need for standardized reporting and analysis of CT findings in pediatric COVID‐19 cases.

Regarding lesion distribution, our study found a predominance of peripheral involvement (45.24% peripheral only, 40.48% both central and peripheral). Notably, combined central and peripheral involvement demonstrated a significant association with consolidation patterns (*p* < 0.001). This pattern aligns with several other studies [23, 25, 28], although some researchers have reported a higher incidence of combined central and peripheral involvement [26]. In adult populations, GGO have been reported in approximately 92.1% of cases and consolidations in about 43% of cases, both predominantly located in the peripheral regions of the lungs [33]. These differences highlight the importance of comprehensive imaging analysis to fully characterize the spatial distribution of COVID‐19‐related lung changes in children.

Lower zone predominance of pulmonary lesions was noted in 69% of cases, with 21.4% showing involvement of the entire lung. This distribution showed strong statistical significance for both ground glass opacities (*p* < 0.001) and consolidation (*p* ≈ 0.002). This finding is consistent with multiple studies that have reported similar patterns of lower lobe predilection in COVID‐19 pneumonia [23, 24, 26]. Similarly, in adult populations, greater involvement of the lower zones has been observed, with the right lower lobe and left lower lobe affected in approximately 85.7% and 82.5% of patients, respectively [33]. Although respiratory syncytial virus (RSV) infection and community‐acquired pneumonia (CAP) can also demonstrate lower lobe predominance [34, 35], in our study, COVID‐19 PCR results were positive, supporting the etiologic diagnosis.

Multi‐lobe involvement was common, with 45.3% of cases showing three or four affected lobes. Notably, cases with less than 25% lung involvement showed significant correlation with ground glass opacities (*p* < 0.001). This extent of involvement is less than that reported in some ICU‐based studies but comparable to findings in general pediatric populations. For instance, Najafinajad et al. [21] reported 47.61% of cases with involvement in all five lobes and 33.35% with involvement in three to four lobes. The differences in lobe involvement patterns between adults and children emphasize the need for age‐specific approaches in radiological assessment and clinical management [36].

Notably, 7.1% of patients in our cohort had normal CT scans, a lower percentage compared to many other studies. This discrepancy might be explained by socioeconomic factors influencing healthcare‐seeking behavior in our region, potentially resulting in later presentations and more advanced disease at the time of imaging. Other studies have reported varying rates of normal CT scans in pediatric COVID‐19 cases, ranging from 0% to 34% [21, 23, 24, 27, 28]. For example, a study in Wuhan, China, found 14% of hospitalized children had no radiological evidence of COVID‐19 [23], while Kumar et al. [27] reported approximately one‐third of children had normal CT scans. Li et al. [37] found 25% of children had no abnormal findings, and Shelmerdine et al. [24] reported 34%. The stark contrast with Najafinejad et al. [21]'s study at the same hospital, which found 0% normal CT scans, further underscores the potential impact of regional socioeconomic factors on disease presentation and severity.

Pleural effusion was uncommon in our study, occurring in only 4.8% of cases. This low incidence is generally consistent with other pediatric studies, although some variations exist across different populations. For instance, Najafinejad et al. [21] reported pleural effusion in 9% of cases at the same hospital. In adult populations, Alian et al. [38] found pleural effusion in 7.6% of patients with bilateral involvement and 7.2% with unilateral involvement in Iran. Interestingly, a systematic review by Shelmerdine et al. [24], which evaluated 421 children, identified only three cases of pleural effusion, further supporting the rarity of this finding in pediatric COVID‐19 cases.

Our study found no cases of mediastinal lymphadenopathy or pericardial effusion. These findings are consistent with the rarity of these complications in pediatric COVID‐19 cases. The systematic review by Shelmerdine et al. [24] also reported no instances of mediastinal lymphadenopathy across the studies they reviewed. However, Bayramoglu et al. [25] found that 8% of patients in their study had mediastinal lymphadenopathy, suggesting some variability in its occurrence. Regarding pericardial effusion, while our study found no cases in children, Yazdi et al. [39] reported a 2% incidence in adult COVID‐19 patients in Iran. Similarly, a meta‐analysis in adults reported that pleural effusion is also very rare, with an incidence of approximately 3.6% [40]. These observations highlight the differences in COVID‐19 manifestations between pediatric and adult populations, emphasizing the need for age‐specific considerations in diagnosis and management.

These findings contribute to the growing body of knowledge of radiological manifestations of COVID‐19 in children. The variations observed across different studies underscore the complex and heterogeneous nature of the disease's presentation in pediatric populations. One of the initial objectives of this study was to conduct follow‐up CT scans several years after the initial diagnosis. However, due to extremely low patient participation, this aspect could not be adequately investigated. Additionally, we recommend that future studies be conducted across multiple centers to provide more comprehensive and generalizable results. Future research should focus on standardizing imaging protocols and reporting methods to facilitate more accurate comparisons across different settings and patient groups. Moreover, the potential influence of socioeconomic factors on disease presentation and severity, as suggested by our findings, warrants further investigation. Understanding these contextual factors could be crucial in developing targeted strategies for early detection and management of COVID‐19 in diverse pediatric populations.

## 5 Conclusion

This study offers valuable insights into the radiological features of COVID‐19 in the pediatric population. The majority of cases presented with GGO and consolidations, while a small percentage (7.1%) showed no visible pulmonary lesions. Peripheral and combined central‐peripheral lung lesions, particularly in the lower lung zones, were commonly observed. Notably, pleural effusion, mediastinal lymphadenopathy, and pericardial effusion were rare, contrasting with findings in adult COVID‐19 cases. About half of the patients exhibited multifocal pulmonary changes involving two to three lung lobes, a pattern that could aid in developing automated diagnostic tools through artificial intelligence. These findings are essential for guiding clinical practices and shaping future research on the radiological characteristics of COVID‐19 in children.

## Author Contributions

**Mohammad Hadi Gharib:** data curation, funding acquisition, resources, software, supervision, visualization, writing – review and editing. **Dayan Amanian:** data curation, project administration, visualization. **Reza Zahedpasha:** conceptualization, formal analysis, investigation, project administration, validation, writing – original draft, writing – review and editing. All authors have read and approved the final version of the manuscript.

## Ethics Statement

Given the retrospective nature of this study, direct patient or public involvement was not required. This study was approved by the Ethics Committee of Golestan University of Medical Sciences https://ethics.research.ac.ir/IR.GOUMS.REC.1403.047.

## Conflicts of Interest

The authors declare no conflicts of interest.

## Transparency Statement

The lead author, Reza Zahedpasha, affirms that this manuscript is an honest, accurate, and transparent account of the study being reported; that no important aspects of the study have been omitted; and that any discrepancies from the study as planned (and, if relevant, registered) have been explained.

## Data Availability

The data sets used and/or analyzed during the current study are available from the corresponding author on reasonable request. Reza Zahedpasha had full access to all of the data in this study and takes complete responsibility for the integrity of the data and the accuracy of the data analysis.
